# Spatial control of the GEN1 Holliday junction resolvase ensures genome stability

**DOI:** 10.1038/ncomms5844

**Published:** 2014-09-11

**Authors:** Ying Wai Chan, Stephen C. West

**Affiliations:** 1 London Research Institute, Cancer Research UK, Clare Hall Laboratories, South Mimms, Herts EN6 3LD, UK

## Abstract

Holliday junction (HJ) resolvases are necessary for the processing of persistent recombination intermediates before cell division. Their actions, however, need to be restricted to the late stages of the cell cycle to avoid the inappropriate cleavage of replication intermediates. Control of the yeast HJ resolvase, Yen1, involves phosphorylation changes that modulate its catalytic activity and nuclear import. Here, we show that GEN1, the human ortholog of Yen1, is regulated by a different mechanism that is independent of phosphorylation. GEN1 is controlled exclusively by nuclear exclusion, driven by a nuclear export signal (NES) that restricts GEN1 actions to mitosis when the nuclear membrane breaks down. Construction of a nuclear-localized version of GEN1 revealed that its premature actions partially suppress phenotypes associated with loss of BLM and MUS81, but cause elevated crossover formation. The spatial control of GEN1 therefore contributes to genome stability, by avoiding competition with non-crossover promoting repair pathways.

## Abstract (web summary)

The human Holliday junction resolvase GEN1 functions during anaphase to eliminate recombination intermediates that block proper chromosome segregation. Here, the authors demonstrate that GEN1 activity is regulated independently of its phosphorylation status and relies on its active exclusion from the nucleus.

## Graphical abstract

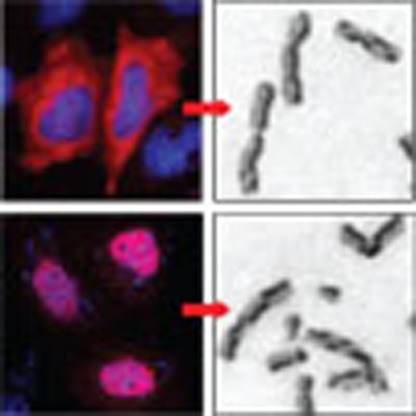

Homologous recombination promotes the repair of DNA double-strand breaks and facilitates the recovery of stalled or collapsed replication folks^1^,^2^. Repair involves a reciprocal exchange of DNA strands between sister chromatids or homologous chromosomes, leading to the formation of intermediates, such as Holliday junctions (HJs), that provide a covalent linkage between sister chromatids or homologous chromosomes^3^,^4^,^5^. The efficient processing of these joint molecules, which is essential for chromosome segregation at cell division, also plays an important role in determining the outcome of recombination: for example, crossovers (COs) between homologous chromosomes are required for meiotic division, whereas non-COs are favored in mitotic cells to avoid loss of heterozygosity^6^,^7^.

In mitotic cells, two major mechanisms ensure the timely elimination of joint molecules. First, the human BTR complex (BLM-TOPIIIα-RMI1-RMI2), or yeast STR complex (Sgs1-Top3-Rmi1), promote the dissolution of double HJs to generate NCO products^8^,^9^. Second, structure-selective endonucleases resolve joint molecules to form both COs and non-COs. These include MUS81-EME1 (refs 10, 11), SLX1-SLX4 (refs 12, 13, 14, 15, 16, 17) and GEN1 (refs 18, 19). MUS81-EME1 and SLX1-SLX4 act in the same pathway, which is distinct from that mediated by GEN1 (refs 15, 16, 20).

It is thought that BTR/STR-mediated dissolution provides the primary pathway for the processing of HJs in mitotic cells since cells derived from individuals with Bloom’s syndrome, that lack BLM activity, exhibit an elevated frequency of sister chromatid exchanges (SCEs) resulting from MUS81-EME1, SLX1-SLX4 and GEN1 mediated HJ resolution^15^,^16^,^20^,^21^. Depletion of the resolvases from either wild type or BLM-deficient cells results in DNA segregation defects^15^,^16^,^17^,^20^,^21^, suggesting that the resolution pathways serve as a safeguard mechanism for the removal of persistent HJs that elude dissolution. The regulation of HJ dissolution/resolution is therefore important for the maintenance of genome stability, both by limiting CO formation and for ensuring proper chromosome segregation.

Recent studies have shown that the enzymatic activities and subcellular localization of the HJ resolvases are tightly controlled and modulated throughout the cell cycle to avoid competition with the dissolution pathway. For example, *Saccharomyces cerevisiae* Mms4 (the ortholog of EME1) is phosphorylated by Cdk and Cdc5 at the G2/M transition, leading to a boost of Mus81-Mms4 nuclease activity^22^,^23^,^24^,^25^. Similarly, in human cells, EME1 is phosphorylated by CDK/PLK1 at prometaphase, but in this case, the modification promotes interactions between MUS81-EME1 and SLX1-SLX4 leading to the formation of an SLX1-SLX4-MUS81-EME1 complex (SLX-MUS) that promotes HJ resolution^15^. The yeast ortholog of GEN1, Yen1, is also regulated by phosphorylation: in S phase, the protein is heavily modified by Cdk-mediated phosphorylation, leading to decreased substrate binding, and additionally, the phosphorylation of S679, which overlaps the nuclear localization signal (NLS), leads to inhibition of its nuclear import^26^,^27^,^28^. Later in the cell cycle, these inhibitory phosphorylation events are reversed by the release/activation of Cdc14 phosphatase, leading to Yen1’s nuclear entry and enzymatic activation. This second boost in resolvase activity processes any remaining HJs and ensures DNA segregation^27^,^28^. Importantly, the presence of nuclear and constitutively active Yen1 (Yen1^ON^) led to increased CO formation, indicating that prematurely activated Yen1 competes with the NCO pathway^27^,^28^. The temporal and spatial restriction of Yen1 activity to the late stages of the cell cycle also appears to be important to protect against the cleavage of replication intermediates that arise during S phase, as premature activation of Yen1 increases the sensitivity of the cells to DNA-damaging agents^27^.

HJ resolution catalyzed by GEN1 is also thought to be restricted to the late stages of the cell cycle^23^, but the underlying mechanism of regulation is unknown. Here, we show that GEN1 is regulated in a manner that is different from that of Yen1. We find that GEN1 contains a powerful nuclear export signale (NES) that mediates its nuclear exclusion such that its actions are restricted until mitosis when the nuclear membrane breaks down. Moreover, when GEN1 is artificially mislocalized to the nucleus an elevated frequency of COs is observed (as indicated by an increased incidence of SCEs), hence increasing the risk of loss of heterozygosity and genomic rearrangements.

## Results

### CRM-mediated nuclear export regulates GEN1 localization

**Figure 1:**
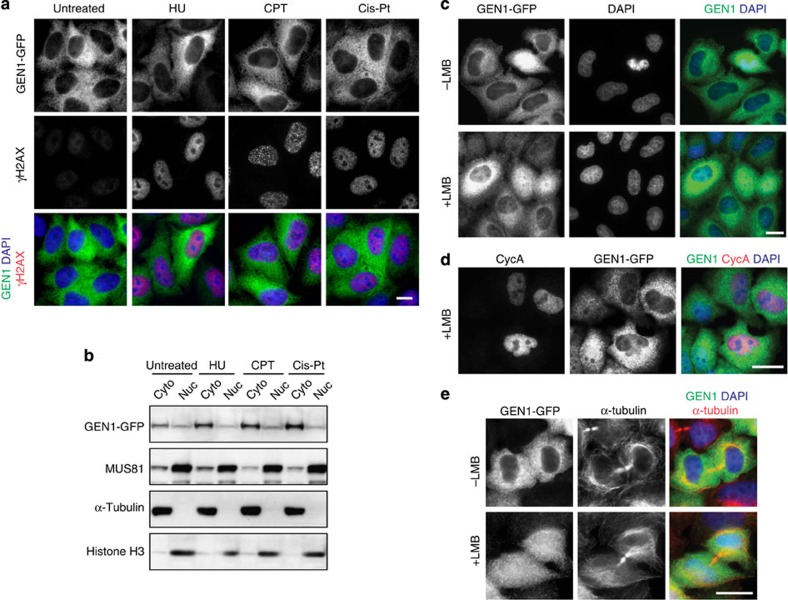
Cellular localization of GEN1. (**a**) HeLa cells expressing GEN1-FLAP were treated with or without hydroxyurea (HU, 2 mM), camptothecin (CPT, 20 nM) or cisplatin (Cis-Pt, 2 μM) for 16 h. GEN1, γH2AX and DNA were visualized using an anti-GFP antibody (green), an anti-γH2AX antibody (red) and DAPI staining (blue), respectively. (**b**) Cell fractionation to determine the localization of GEN1. Fractions were analysed by western blotting for the indicated proteins. (**c**) HeLa cells expressing GEN1-FLAP were treated with or without 25 nM LMB for 4 h. GEN1 and DNA were visualized as in **a**. (**d**) As **c**, except the LMB-treated cells were stained for Cyclin A (red) to identify S- and G2-phase cells. (**e**) As **a**, with α-tubulin staining decorating the mid-body and marking late mitotic cells. Scale bars, 10 μm.

Previous studies have shown that GEN1 is cytoplasmic throughout interphase^23^,^29^. However, because the subcellular localization of many DNA repair-related proteins (e.g. p53, p38MAPK) is affected by DNA damage^30^,^31^, we used FLAP (GFP and FLAG)-tagged GEN1, expressed at endogenous levels from a bacterial artificial chromosome^23^, to determine whether the localization of GEN1 is altered by treatments that either block replication fork progression (hydroxyurea) or introduce DNA lesions (camptothecin, cisplatin). In all cases, we found that GEN1 localized mainly to the cytoplasm (Fig. 1a). These results were confirmed by cellular fractionation, which led us to estimate that ~80% of the GEN1 was present in the cytoplasmic fraction of both untreated and treated cells, with the remaining 20% in the nuclear fraction (Fig. 1b). These results indicate that the bulk of the GEN1 protein gains access to DNA only after the breakdown of the nuclear envelope at mitotic entry. In contrast, MUS81 localized mainly to the nucleus (Fig. 1b), indicating that the actions of the two resolvases are spatially distinct in interphase cells.

To determine whether GEN1 localization might be controlled by the nuclear export machinery, we used leptomycin B (LMB) to inhibit CRM1, a major nuclear export receptor^32^,^33^. After treatment with LMB, a proportion of the interphase cells (23.7±1.5%, *n*>350 cells) exhibited a clear increase in the nuclear localization of GEN1 (Fig. 1c). As not all cells showed this increase in response to LMB treatment, we determined whether the effect was specific to the stage of the cell cycle by staining for cyclin A, which serves as a marker for S/G2 cells. We found that ~47% of the G1 cells (that is cyclin A negative) showed nuclear accumulation of GEN1 on LMB treatment (*n*=236 cells). In contrast, none of the S/G2 cells responded to LMB treatment (*n*=169 cells). These results led us to conclude that the cells responding to LMB treatment and exhibiting nuclear GEN1 after LMB treatment were in G1 (Fig. 1d). As the nuclear envelope reforms at the end of mitosis, we speculated that nuclear export of GEN1 must occur at telophase, and that LMB might specifically block the transport of GEN1 to the cytoplasm at this stage of the cell cycle. This was indeed shown to be the case because, in the absence of LMB, GEN1 was excluded from the nucleus in all telophase cells examined (*n*=102 cells) as detected by α-tubulin staining of the mid-body, whereas almost all telophase cells exhibited nuclear staining for GEN1 following LMB treatment (109 out of 112 telophase cells) (Fig. 1e).

### Effect of phosphorylation on GEN1 activity and function

**Figure 2:**
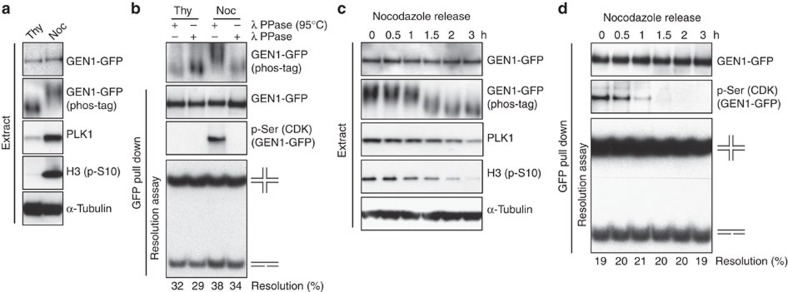
GEN1 activity is independent of its phosphorylation status. (**a**,**b**) Extracts were prepared from HeLa cells expressing GEN1-FLAP after synchronization with thymidine (Thy) or nocodazole (Noc), as indicated. Cell extracts were analysed by western blotting for the indicated proteins (**a**). GEN1-FLAP was affinity purified from each sample by GFP pull down, treated with active or heat-inactivated λ-phosphatase (95 °C), and assayed for HJ resolution activity (**b**). Phosphorylated GEN1 was detected using an anti-phosphoserine antibody (p-Ser) that is specific for CDK substrates. (**c**,**d**) Extracts were prepared from HeLa cells expressing GEN1-FLAP after release from nocodazole arrest at the indicated time points. Cell extracts were analysed by western blotting for the indicated proteins (**c**). GEN1-FLAP was affinity purified from each sample by GFP pull down and assayed for HJ resolution activity (**d**).

The cellular localization and substrate binding activities of yeast Yen1 are directly controlled by changes to its phosphorylation status^27^,^28^. To determine whether GEN1 is also regulated by phosphorylation during the mitotic cell cycle, cells were synchronized at S phase (using a thymidine block) and at prometaphase (by nocodazole shake-off), respectively. Mitotic arrest was indicated by the accumulation of PLK1 and the phosphorylation of histone H3 at S10 (Fig. 2a). A phosphorylated form of GEN1 was detected at M phase, as determined by its reduced electrophoretic mobility on phos-tag gels (Fig. 2a,b), and by western blotting using an antibody specific for phospho-(Ser) CDK substrates (Fig. 2b). However, when GEN1 was affinity purified from the S- and M-phase extracts, and assayed for its ability to resolve HJs, we did not observe any great difference in the levels of activity, although slightly more activity was found in the M-phase samples (38% resolution versus 32%). Moreover, the activities were largely unaffected by treatment with λ-phosphatase that promotes GEN1’s dephosphorylation (Fig. 2b). The phosphorylation of GEN1 was reduced at mitotic exit, as indicated by its increased electrophoretic mobility (Fig. 2c) and the disappearance of the phosphoserine signal (Fig. 2d), but its HJ resolving activity remained relatively constant during mitotic exit (Fig. 2d). Similarly, when cells were synchronized by a double thymidine block followed by release, we again found that GEN1 activity was relatively constant during cell cycle progression from G1/S phase to mitosis (Supplementary Fig. 1).

**Figure 3:**
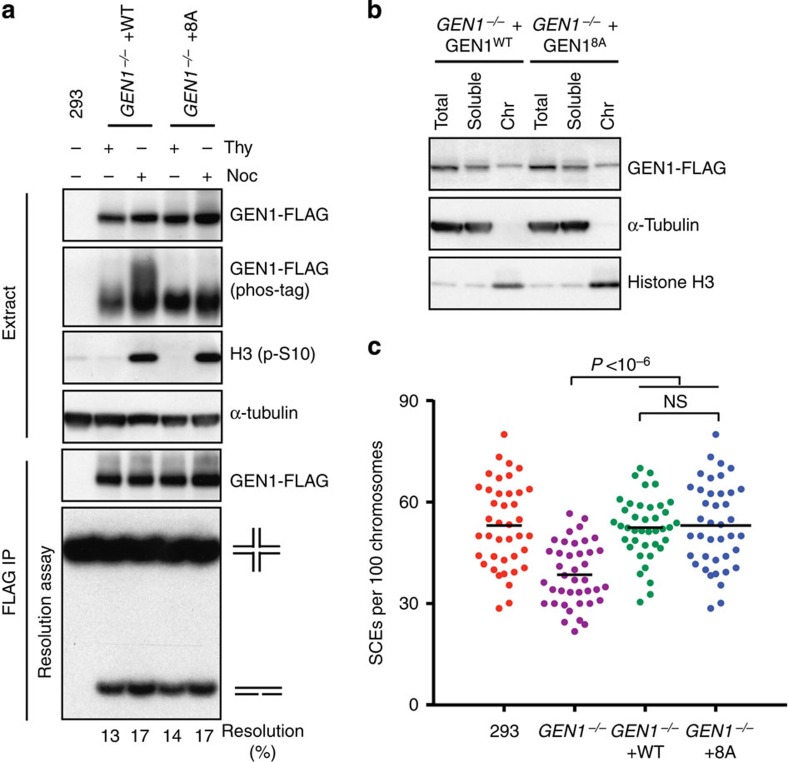
Functional activity of a GEN1 phosphomutant. (**a**) Extracts were prepared from *GEN1*^*−/−*^ Flp-In T-REx 293 cells expressing GEN1^WT^ or GEN1^8A^ after synchronization with Thymidine (Thy) or Nocodazole (Noc) as indicated. Cell extracts were analysed by western blotting for the indicated proteins. GEN1-FLAG was affinity purified from each sample using anti-FLAG M2 agarose beads and assayed for HJ resolution activity. Parental Flp-In T-Rex 293 cells (293) were used as a control. (**b**) Biochemical fractionation of *GEN1*^*−/−*^ cells expressing GEN1^WT^ or GEN1^8A^ after overnight synchronization with Nocodazole. The whole-cell extract (total), soluble and chromatin (Chr) fractions were analysed by western blotting for the indicated proteins. (**c**) Flp-In T-Rex 293 cells, *GEN1*^*−/−*^ cells and *GEN1*^*−/−*^ cells expressing GEN1^WT^ or GEN1^8A^ were treated with cisplatin (2 μM) for 1 h. SCE formation was quantified as described. Forty metaphase cells (>1,600 chromosomes) were counted per condition. Black bars represent the mean number of SCEs per 100 chromosomes per spread. *P* values were determined using a two-tailed *t*-test. NS: not significant.

In yeast, Yen1 is phosphorylated by Cdk^27^. To determine whether CDK phosphorylation affects GEN1 activity, all putative CDK sites (S/TP) in GEN1 were mutated to alanine (S249A, S269A, S512A, T535A, S598A, S655A, S842A and S893A). Previously, the phosphorylation of one of these CDK sites (S893) was identified in proteomic studies^34^,^35^. The actions of the resulting phosphomutant, GEN1^8A^, together with GEN1 as a control, were then analysed in a GEN1 knockout cell line (*GEN1*^*−/−*^) generated using the CRISPR-Cas9 system^36^,^37^ that targeted exon 2 of *GEN1* locus (Supplementary Fig. 2a). The knockout was verified by both sequencing (Supplementary Fig. 2b,c) and western blotting (Supplementary Fig. 2d). We found that GEN1^8A^ did not show a reduced electrophoretic mobility on nocodazole-induced mitotic block (Fig. 3a), indicating that the phosphorylation of GEN1 is mediated primarily by mitotic CDK. Furthermore, when GEN1^WT^ and GEN1^8A^ were affinity purified from the S- and M-phase extracts and assayed for activity (Fig. 3a), they exhibited similar levels of HJ resolution activity.

To determine whether CDK-mediated phosphorylation of GEN1 modulates its chromatin binding, cells were synchronized by overnight treatment of nocodazole followed by biochemical fractionation into separate soluble and chromatin fractions (Fig. 3b). We found that the same amounts of GEN1^WT^ and GEN1^8A^ were found in the chromatin fraction, arguing against the notion that phosphorylation regulates GEN1-chromatin interaction. Finally, we compared the SCE levels of cells expressing GEN1^WT^ and GEN1^8A^. As expected, *GEN1*^*−/−*^ cells showed a significant reduction of cisplatin-induced SCEs when compared with the parental wild-type cells (Fig. 3c), but this reduction was fully rescued by both GEN1^WT^ and GEN1^8A^ (Fig. 3c). Taken together, these results show that the temporal restriction of GEN1 function is achieved primarily through CRM1-mediated nuclear export, rather than by the modulation of GEN1 activity by changes to its phosphorylation status. These results are in stark contrast to the mechanisms by which Yen1 is regulated in yeast^27^,^28^.

### Identification of a nuclear export signal in GEN1

**Figure 4:**
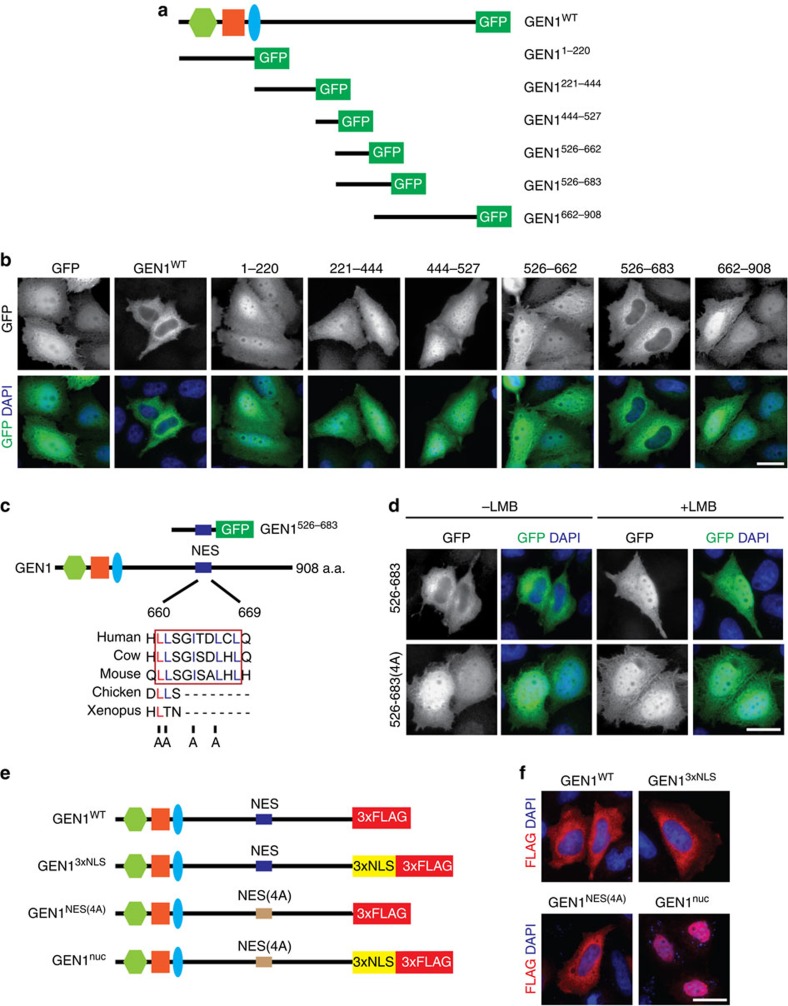
Cytoplasmic localization of GEN1 is directed by a nuclear export signal. (**a**) Schematic representation of a series of GEN1 truncations. The green hexagon, the orange square and the blue ellipse represent the XPG-N, XPG-I and the helix–hairpin–helix domains, respectively. GFP signifies the C-terminal tag. (**b**) HeLa cells, transfected with the GEN1-GFP tagged constructs, or GFP alone, were visualized by immunofluorescence. GEN1-GFP and DNA were visualized as in Fig. 1. (**c**) Schematic representation of the putative nuclear export signal (NES) of GEN1, which is conserved between various mammalian species. (**d**) HeLa cells, transfected with GEN1^526–683^-GFP or GEN1^526–683(4A)^-GFP, were treated with or without LMB. GEN1 and DNA were visualized as above. (**e**) Schematic representation of GEN1^WT^, GEN1 with 3xNLS sequences (GEN1^3xNLS^), GEN1 with mutated NES (GEN1^NES(4A)^) and GEN1 with a mutated NES and 3xNLS sequences (GEN1^nuc^). (**f**) HeLa cells, transfected with the GEN1 constructs shown in **e**, were analysed for GEN1-FLAG and DNA using an anti-FLAG antibody and DAPI staining, respectively. Scale bars, 10 μm.

CRM1 mediates nuclear export by interacting with the NES of its cargo proteins^33^, leading us to attempt to map the location of a NES in GEN1. To do this, a series of GEN1 truncations, fused in-frame with GFP, were generated (Fig. 4a) and the cellular localization of each expressed protein was determined (Fig. 4b). The small size of each truncation protein was expected to allow nuclear entry by passive diffusion through nuclear pores^38^, such that the NES-containing fragment of GEN1 could be identified by its nuclear exclusion. As observed with GFP alone, most of the truncations localized both in the cytoplasm and the nucleus, except for GEN1^526–683^-GFP that displayed a clear cytoplasmic localization (Fig. 4b). Examination of the sequence of this construct revealed a leucine-rich sequence (660-LLSGITDLCL-669) (Fig. 4c), conforming to the NES consensus sequence (L-x(2-3)-[LIVFM]-x(2-3)-L-x-[LI])^39^.

When cells expressing GEN1^526–683^ were treated with LMB we found that nuclear exclusion of the polypeptide was inhibited (Fig. 4d). Similarly, mutation of four leucine/isoleucine residues within the presumed NES consensus (4A: L660A, L661A, I664A and L667A) abolished the nuclear exclusion of GEN1^526–683^, confirming the presence of a functional NES (Fig. 4d). Interestingly, attempts to overcome the NES by generating a GEN1 fusion protein containing three nuclear localization signals (3xNLS) were insufficient to induce its relocalization to the nucleus (Fig. 4e,f). However, we were successful in driving the nuclear localization of GEN1 by adding three NLS sequences and by mutating the four leucine/isoleucine residues in the NES (Fig. 4e,f). In this case, GEN1 was found to be constitutively nuclear, and in the following work, we will refer to this as GEN1^nuc^.

### Nuclear localization of GEN1 promotes crossover formation

**Figure 5:**
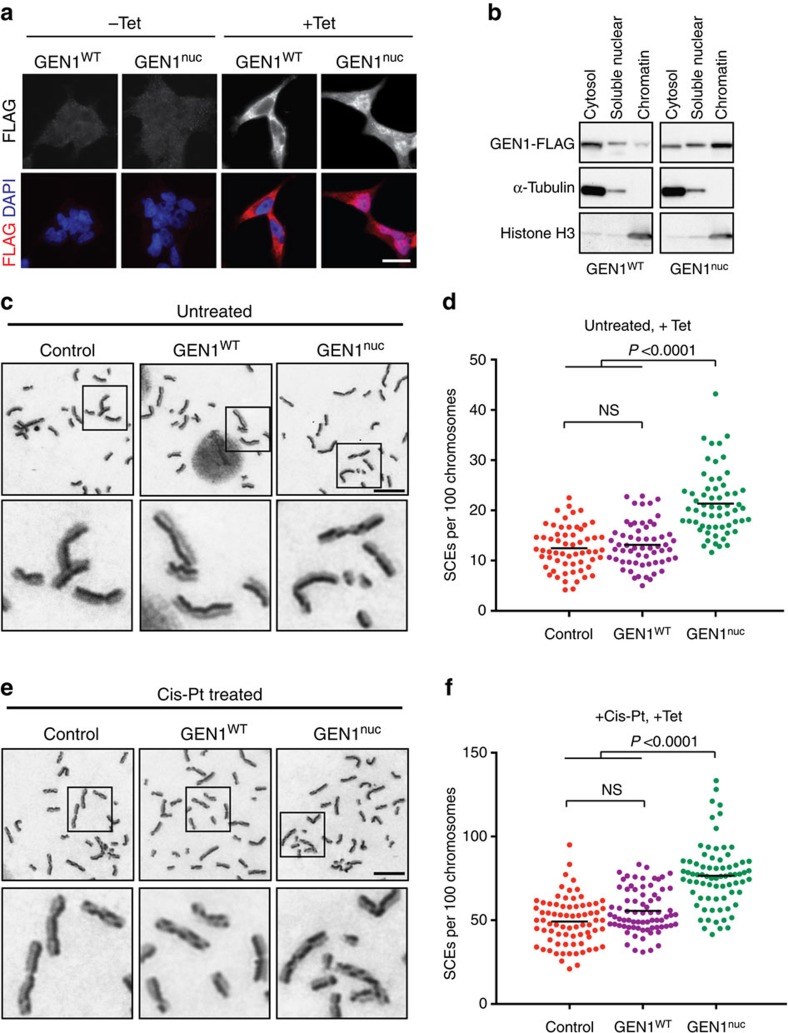
Expression of nuclear GEN1 leads to elevated SCE formation. (**a**) Flp-In T-REx 293 cells stably expressing GEN1^WT^ or GEN1^nuc^ were treated with or without tetracycline (Tet) for 48 h. GEN1 and DNA were visualized using an anti-FLAG antibody and DAPI staining, respectively. Scale bar, 10 μm. (**b**) Biochemical fractionation of cells expressing GEN1^WT^ or GEN1^nuc^ after overnight synchronization with hydroxyurea (2 mM). The fractions were analysed by western blotting for the indicated proteins. (**c**) Representative images of metaphase spreads from parental Flp-In T-REx 293 cells (control), and cells expressing GEN1^WT^ or GEN1^nuc^. (**d**) Quantification of SCE formation, as determined in **c**. Each data point represents a single metaphase. 60 metaphase cells (>2,600 chromosomes) were counted per condition. Black bars represent the mean number of SCEs per 100 chromosomes per spread. *P* values were determined using a two-tailed *t*-test. (**e**) As **c**, except the cells were treated with cisplatin (2 μM). (**f**) Quantification of SCE formation, as in **e**.

In contrast to yeast Yen1, GEN1 nuclease activity does not appear to be regulated by phosphorylation during the cell cycle, leading us to reason that forced nuclear import of GEN1 would lead to genome instability due to its premature action. To study the effect of GEN1 deregulation, we generated stable HEK293 cells expressing wild-type GEN1 (GEN1^WT^) or GEN1^nuc^ under the control of a tetracycline-inducible promoter. Both proteins carried carboxy-terminal 3xFLAG tags that allowed us to visualize GEN1 expression by immunostaining. As expected, GEN1^nuc^ was enriched in the nucleus, whereas GEN1^WT^ was mainly cytoplasmic (Fig. 5a). To determine whether nuclear GEN1 was chromatin bound, cells were arrested in S phase using hydroxyurea followed by biochemical fractionation. We found that a significant amount of the GEN1^nuc^ was associated with the chromatin fraction in these S-phase cells (Fig. 5b). Importantly, affinity purified GEN1^nuc^ from these extracts displayed a similar HJ resolvase activity as GEN1^WT^, showing that inclusion of the 3xNLS sequence and mutation of the NES did not affect protein activity (Supplementary Fig. 3a).

To determine whether expression of GEN1^nuc^ leads to an increased sensitivity to genotoxic stress, cells expressing GEN1^WT^ or GEN1^nuc^ were treated with various concentrations of hydroxyurea, camptothecin or cisplatin and analysed for colony formation. We found that GEN1^nuc^ did not affect cell survival (Supplementary Fig. 3b). Moreover, expression of GEN1^nuc^ failed to induce an increase in the levels of phosphorylated H2AX (γH2AX) nor affect the DNA damage checkpoint response, measured by the phosphorylation of CHK1 at S345 and KAP-1 at S842, either in untreated or damaged cells (Supplementary Fig. 3c). These results indicate that premature nuclear import of GEN1 is not particularly harmful to the cells.

One predicted effect of GEN1^nuc^ is that it would be expected to compete with BTR-mediated HJ dissolution and thereby promote mitotic CO formation. We therefore examined SCE levels in both untreated cells (Fig. 5c, and quantified in Fig. 5d) and those treated briefly with cisplatin to elevate the level of recombination intermediates (Fig. 5e and quantified in Fig. 5f). In both cases, cells expressing GEN1^nuc^ exhibited significantly increased SCE formation, compared with control cells or cells expressing GEN1^WT^ (Fig. 5c–f; Supplementary Fig. 4a shows the expression levels of GEN1). As controls, cells that were not treated with tetracycline (thus no GEN1 induction) showed no significant difference in SCE levels (Supplementary Fig. 4b). Taken together, these results show that restriction of GEN1 action (by nuclear exclusion) to the late stages of the cell cycle is critical for preventing mitotic CO formation.

### GEN1^nuc^ suppresses BLM and MUS81 depletion phenotypes

Persistent unresolved HJs induce various cellular defects including chromosome breakage and aberrant chromosome segregation^15^,^17^,^20^,^21^. We therefore determined whether expression of nuclear GEN1 would suppress defects in other HJ processing pathways. To do this, BLM, MUS81 or BLM and MUS81 were small interfering RNA (siRNA)-depleted from cells expressing GEN1^WT^ or GEN1^nuc^ from a tetracycline-inducible promoter. All proteins were depleted with a high efficiency (Supplementary Fig. 4c), and GEN1^WT^ or GEN1^nuc^ were induced at comparable levels in the siRNAs-treated cells (Supplementary Fig. 4d).

**Figure 6:**
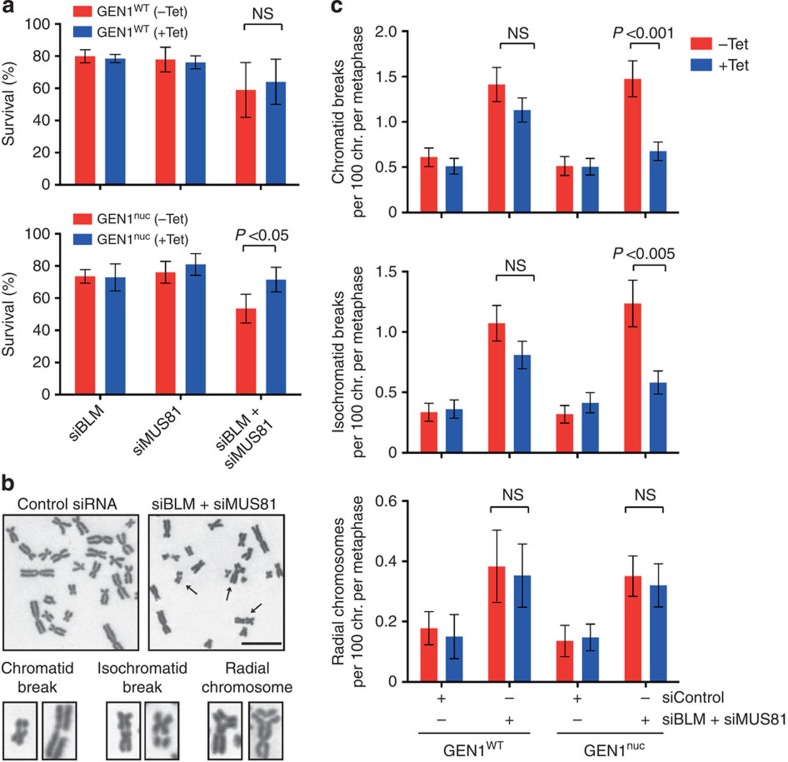
Rescue of BLM/MUS81 defects by expression of GEN1^nuc^. (**a**) Clonogenic cell survival assays were carried out on Flp-In T-REx 293 cells expressing GEN1^WT^ or GEN1^nuc^ (±Tet) and depleted for the indicated proteins. Cell survival after treatment with the control siRNA is defined as 100%. The data represent the mean±s.d. of at least three independent experiments. *P* values were determined using a two-tailed *t*-test. (**b**) Representative images of metaphase spreads of Flp-In T-REx 293 cells treated with the indicated siRNAs. Three types of chromosome aberrations (chromatid breaks, isochromatid breaks and radial chromosomes) were identified and quantified. (**c**) Flp-In T-REx 293 cells expressing GEN1^WT^ or GEN1^nuc^ (±Tet) were treated with the indicated siRNAs, and metaphase spreads were analysed and quantified for the indicated chromosome aberrations. One-hundred twenty metaphase cells (>5,500 chromosomes) were analysed. The data represent the mean number of aberrations per 100 chromosomes per spread±s.e.m. *P* values were determined using a two-tailed *t*-test.

First, we determined the effect of GEN1^WT^ or GEN1^nuc^ expression on cell viability using clonogenic survival assays. As expected, cells depleted of both MUS81 and BLM exhibited a significant loss of viability, and this was partially rescued by expression of GEN1^nuc^ but not GEN1^WT^ (Fig. 6a). Second, we determined whether GEN1^nuc^ could suppress the high levels of chromosome breakage seen in metaphase spreads from BLM and MUS81 co-depleted cells^17^,^21^. In these experiments, we scored several types of chromosome aberrations, including chromatid breaks (only one arm of the chromosome is broken), isochromatid breaks (both arms are broken) and radial chromosomes (Fig. 6b). Importantly, we found that expression of GEN1^nuc^, but not GEN1^WT^, rescued the increased frequencies of chromatid and isochromatid breaks (Fig. 6c, upper and center panels). In contrast, there was little effect on the frequency of radial chromosomes (Fig. 6c, lower panel), indicating that these do not arise from a loss of HJ processing activity. Together, these results show that the premature actions of nuclear GEN1 lead to the removal of recombinant intermediates that are normally processed earlier in the cell cycle, either by the BTR complex or by MUS81-EME1.

## Discussion

**Figure 7:**
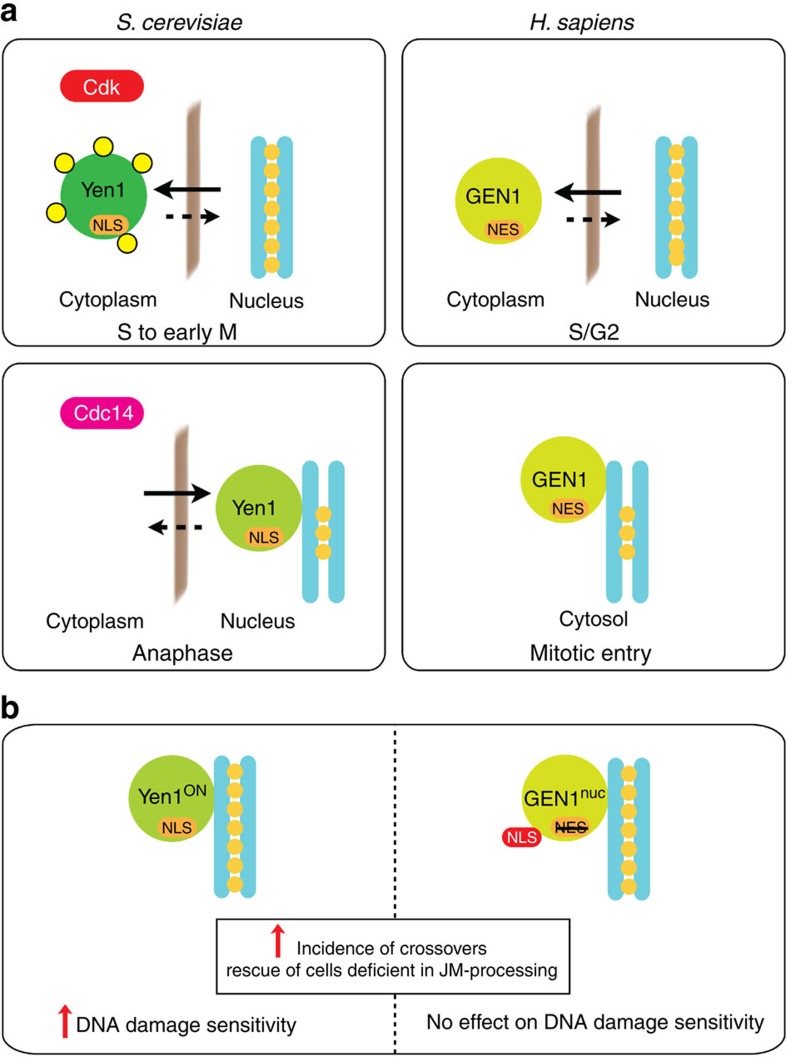
Distinct mechanisms of regulatory control for *S. cerevisiae* Yen1 and human GEN1. (**a**) Regulatory control mechanisms (see text for details). (**b**) Phenotypic effects of expressing Yen1^ON^ and GEN1^nuc^ (see text for details).

In this work, we have shown that the activity of the GEN1 Holliday junction resolvase is restricted to mitosis by nuclear exclusion. The mechanism of GEN1 regulation contrasts with that observed in *S. cerevisiae*, where the GEN1 ortholog Yen1 undergoes a dual mechanism of regulation^26^,^27^,^28^. In yeast, Cdk-mediated phosphorylation of Yen1, which takes place during S phase, promotes its nuclear exclusion and inhibits its catalytic activity. Nuclear exclusion of Yen1 is achieved by: (i) impairing its nuclear import by phosphorylation of S679, which overlaps the NLS, and (ii) Msn5-mediated nuclear export. S-phase phosphorylation also directly inhibits the nuclease activity of Yen1 by reducing its DNA binding affinity^27^. At the onset of anaphase, however, Cdc14 phosphatase interacts with Yen1 and promotes its dephosphorylation, triggering both its nuclear import and an increase of its DNA binding affinity. In human cells, the action of GEN1 is also restricted in mitosis to avoid competition with the BTR-mediated NCO pathway, although the underlining control mechanism is distinct from that of Yen1. We find that GEN1 was phosphorylated at M phase primarily by CDK. However, the phosphorylation status of GEN1 did not appear to have a significant effect on its nuclease activity or ability to bind chromatin. Moreover, we found that the nonphosphorylatable mutant of GEN1 (GEN1^8A^) was equally active as the wild type in complementing GEN1 knockout cells in terms of SCE formation. The nuclear exclusion of GEN1 appears to be mainly responsible for regulating the activity of GEN1, and is mediated by a NES located within the C-terminal region of the protein. On entry into mitosis, breakdown of the nuclear envelope allows the binding of GEN1 to chromatin. Finally, at mitosis, when the nuclear envelope reforms, GEN1 again becomes excluded from nucleus. A comparison of the mechanisms of regulation of Yen1 and GEN1 is shown in Fig. 7a.

Why have lower and higher eukaryotes evolved different mechanisms of Yen1/GEN1 regulation? One explanation is that yeast undergoes a ‘closed’ mitosis in which the nuclear envelope remains intact. As a consequence, active import machineries have to be employed to shuttle Yen1 into the nucleus. In contrast, in human cells, the physical barrier is automatically disassembled at the G2/M transition, so that cells only need to maintain GEN1 export to prevent its accumulation in the nucleus. Also, again in contrast to the situation in yeast, the human CDC14 orthologs CDC14A and CDC14B do not appear to play essential roles in the reversal of mitotic CDK-mediated phosphorylation to control mitotic exit^40^,^41^.

The consequences of the nuclear presence of deregulated Yen1/GEN1 have been revealed in studies with both Yen1^ON^ and GEN1^nuc^, as summarized in Fig. 7b. Expression of Yen1^ON^ (in which all nine Cdk consensus sites were mutated to alanine) and GEN1^nuc^ led to an increased CO frequency, demonstrating that deregulated Yen1/GEN1 competes with the non-crossover repair pathway^27^,^28^. However, while expression of Yen1^ON^ caused a cellular hypersensitivity to genotoxic stress^27^, we did not observe increased DNA damage sensitivity or a checkpoint response in cells expressing GEN1^nuc^. One possibility that may account for this difference is the broader substrate specificity exhibited by Yen1 in comparison with GEN1 (M. G. Blanco, Y. W. C. and S. C. W., unpublished data). Indeed, the actions of deregulated/nuclear Yen1 may damage DNA intermediates formed during replication fork regression, leading to a hypersensitivity to DNA-damaging agents that promote replication fork stalling. Further studies of the biochemical properties of Yen1 and GEN1 will be required before we gain a more complete understanding of the actions of these flap/fork endonucleases on recombination/replication intermediates.

Our work shows that mislocalization of GEN1 to the nucleus increases CO formation that may in turn promote loss of heterozygosity. This raises the possibility that uncontrolled GEN1 activity could be linked to tumorigenesis. Interestingly, using plasmid-based systems, it was recently shown that GEN1 can act on palindromic AT-rich repeats identified on human chromosomes 11 and 22, that extrude to form cruciform-like structures, giving rise to translocation-like rearrangements^42^,^43^. Carriers of chromosomal translocations t(11,22)(q23;q11), caused by those palindromic AT-rich repeats, may give birth to offspring manifesting Emanuel syndrome^44^. However, at the present time, no cancer/disease-associated mutations have been identified in the NES region of GEN1, so it is not presently clear whether GEN1 misregulation is a driver for disease development.

Taken together, our work provides the first insights into the spatial regulation of GEN1, which serves at least two purposes: First, to ensure the removal of persistent HJs that have escaped the dissolution pathway before cell division and thereby safeguard chromosome segregation. This may be particularly important when cells are exposed to DNA-damaging agents, or replication stress, which leads to an increase in recombination events that could ‘overload’ the BTR complex. Second, to prevent competition with the dissolution pathway mediated by the BTR complex, therefore avoiding mitotic CO formation that can lead to loss of heterozygosity and genomic rearrangements.

## Methods

### Plasmids

The GEN1 truncation constructs (full-length GEN1, GEN1^1–220^, GEN1^221–444^, GEN1^444–527^, GEN1^526–662^, GEN1^526–683^ and GEN1^662–908^) were generated by PCR and cloned in-frame into a modified pDONR221 vector (Life Technologies) encoding a C-terminal GFP tag using an In-Fusion HD cloning kit (Clontech). The coding sequences were then shuttled by Gateway recombination (Life Technologies) into a modified pcDNA5/FRT vector (Life Technologies) with a Gateway cassette RfB cloned into the EcoRV site. To generate GEN1^nuc^, a 3xNLS sequence (DPKKKRKVDPKKKRKVDPKKKRKV) and a 3xFLAG tag sequence (AGDYKDHDGDYKDHIDYKDDDDK) were added to the C terminus of GEN1 by PCR. GEN1 was then cloned into a pcDNA5/FRT/TO vector (Life Technologies). The NES of GEN1 (4A: L660A, L661A, I664A, L667A) and eight putative CDK sites (8A: S249A, S269A, S512A, T535A, S598A, S655A, S842A and S893A) were mutated using QuikChange Lightning Multi Site-Directed Mutagenesis Kit (Agilent). To generate the sgRNA vector for *GEN1* targeting, a pair of annealed oligos (5′-CACCGCACATCCCCTTGCGTAATCT 3′ and 5′ AAACAGATTACGCAAGGGGATGTGC-3′) were cloned into the pX330 plasmid^36^ digested with BbsI according to published protocols^37^.

### Cell culture and transfection

HeLa Kyoto cells, HeLa Kyoto cells expressing GEN1-FLAP from a BAC^23^ and Flp-In T-REx 293 cells were cultured at 37 °C in 5% CO_2_ in DMEM (Life Technologies) supplemented with 10% fetal bovine serum (Life Technologies) and penicillin/streptomycin. Hygromycin (200 μg ml^−1^), zeocin (50 μg ml^−1^) and blasticidin (4 μg ml^−1^) were obtained from Life Technologies. Nocodazole (100 ng ml^−1^), thymidine (2 mM), cisplatin, hydroxyurea, camptothecin and BrdU (100 μM) were obtained from Sigma-Aldrich. Colcemid (0.2 μg ml^−1^) was obtained from Roche. Leptomycin B (25 nM) was obtained from Santa Cruz. HeLa cells were transfected with plasmids encoding various GEN1 truncations for 30 h using TransIT-2020 (Mirus Bio LLC). To generate GEN1-expressing stable cell lines, Flp-In T-REx 293 cells were co-transfected with plasmids encoding the GEN1 proteins and the Flp recombinase encoding plasmid pOG44 (in 1:9 ratio) using FuGENE (Promega). Hygromycin-resistant colonies were picked and expanded. The expression of GEN1^WT^ and GEN1^8A^ were induced with 0.1 μg ml^−1^ tetracycline (Sigma-Aldrich). The expression of GEN1^nuc^ was induced with 1 μg ml^−1^ tetracycline.

To generate the *GEN1*^*−/−*^ cell line, Flp-In T-REx 293 cells were transfected with pX330 cloned with the *GEN1* targeting sequence, along with pSuper.puro (Oligoengine) at a 9:1 ratio. After 24–48 h, cells were selected with 5 ug ml^−1^ puromycin, and seeded as single colonies. Clones were picked and first selected on the basis of a negative signal when western blotted for GEN1. Selected clones were then verified by sequencing. To do this, genomic DNA was extracted from cells with DNeasy Blood & Tissue kit (Qiagen), and PCR was carried out with KOD Hot Start DNA Polymerase (Novagen) to amplify the targeted locus using a forward (5′-TGGCTTATAATATATTGTTTG-3′) and a reverse (5′-GCTTTTAGTATCTGAAGCATC-3′) primer. The PCR product was then purified by agarose gel electrophoresis followed by gel purification with QIAquick Gel Extraction kit (Qiagen) and finally sequenced.

### Lysate preparation and HJ resolution assay

Cell lysates were prepared by suspending cells in lysis buffer (50 mM Hepes pH 7.4, 150 mM NaCl, 0.1% Triton X-100, 10% glycerol, 1 mM DTT) supplemented with protease and phosphatase inhibitors. The lysates were passed through a 0.8 × 40 mm needle (20 × ) followed by incubation on ice for 30 min. The lysates were then cleared by centrifugation (14,000 r.p.m., 30 min). For HJ resolution assays, tagged GEN1 was affinity purified from the cleared lysates using anti-FLAG M2 agarose beads (Sigma-Aldrich) or GFP-Trap beads (Chromotek). The beads were washed extensively with lysis buffer and then mixed with 10 μl of cleavage buffer (50 mM Tris–HCl pH 7.5, 1 mM MgCl_2_, 1 mM DTT) and ~1 nM 5′-^32^P-end-labelled HJ X0 DNA^18^. After 10 min of incubation at 37 °C, DNA products were deproteinized for 45 min at 37 °C by addition of 2.5 μl stop buffer (100 mM Tris–HCl pH 7.5, 50 mM EDTA, 2.5% SDS and 10 mg ml^−1^ proteinase K). Loading buffer was added and radiolabelled products were separated by 10% neutral polyacrylamide gel electrophoresis, and analysed by autoradiography or by phosphorimaging using a Typhoon scanner and ImageQuant software (GE Healthcare).

### Dephosphorylation assay

GEN1-FLAP was affinity purified with GFP-Trap beads. The beads were then incubated with λ-phosphatase (NEB) or λ-phosphatase that had been inactivated by heating for 10 min at 95 °C. The reaction mixtures were then incubated at 30 °C for 20 min before washing extensively with lysis buffer and analysed by western blotting and for HJ resolution.

### Western blotting

Western blotting was carried out as described^23^. Phos-tag SDS–polyacrylamide gel electrophoresis gels were made from 7% acrylamide (37.5:1 crosslinking ratio), 70 μM Phos-tag reagent (Wako Pure Chemical Industries) and 140 μM MnCl_2_, and were processed according to the manufacturer’s manual. Proteins were detected using the following primary antibodies: mouse anti-GFP (1:1,000, Roche 11814460001), mouse anti-PLK1 (1:2,000, Santa Cruz sc-17783), mouse anti-histone H3 pSer10 (1:5000, Abcam 14955), mouse anti-α-tubulin (1:5000, Sigma-Aldrich T9026), rabbit anti-phospho-(Ser) CDK substrate (1:1000, Cell Signaling 2324), rabbit anti-histone H3 (1:2000, Abcam ab1791), mouse anti-FLAG HRP (1:1,000, Sigma-Aldrich A8592), mouse anti-MUS81 (1:1,000, Santa Cruz sc-47692), mouse anti-BLM (1:500, Santa Cruz sc-70426), rabbit anti-KAP-1 pSer842 (1:1000, Abcam ab70369), rabbit anti-CHK1 pSer345 (1:1000, Cell Signaling 2341), mouse anti-histone H2A.X pSer139 (1:1000, Millipore 05-636-1), rabbit anti-GEN1 (1:100, raised against GEN1^890–908^)^19^. Uncropped blots are shown in Supplementary Fig. 5.

### Immunofluorescence

HeLa Kyoto cells or Flp-In T-REx 293 cells were fixed and processed for immunofluorescence as described^45^. In brief, cells were fixed in 4% paraformaldehyde for 10 min at the room temperature followed by ice-cold methanol for 1 min. The cells were then wash with 1 × phosphate-buffered saline (PBS) and permeabilized with 0.5% Triton X-100 for 5 min before antibody staining. The primary antibodies used were as follows: mouse anti-histone H2AX pSer139 (1:1,000, Millipore 05-636-1), mouse anti-α-tubulin (1:5,000, Sigma-Aldrich T9026), rabbit anti-GFP (1:5,000, Abcam ab290), mouse anti-cyclin A (1:200, Santa Cruz sc-56299), mouse anti-FLAG (1:1,000, Sigma-Aldrich A8592). Secondary antibodies conjugated to Alexa Fluor 488 and Alexa Fluor 555 (1:1,000, Molecular Probes) were used for detection. DNA was stained with 4,6-diamidino-2-phenylindole. Images were acquired using Zeiss AXIO Imager M1 with a × 40 EC-Plan-Neofluor lens and Hamamatsu photonics camera under the control of Volocity software. Images were processed using Adobe Photoshop.

### Subcellular fractionation

Cells were collected and washed with PBS and suspended in hypotonic buffer (50 mM Tris–HCl pH 7.5, 10 mM NaCl, 1.5 mM MgCl_2_, 0.34 M sucrose, 10% glycerol and protease and phosphatase inhibitors). Cells were lysed by passing through a 0.8 × 40 mm needle (10 × ) followed by incubation on ice for 15 min. Triton X-100 was added to a final concentration of 0.1%, and the cells were incubated on ice for 5 min. The soluble cytoplasmic fraction and nuclear pellet were collected by centrifugation at 1,300 *g* for 4 min. The cytoplasmic fraction was spun at 20,000 *g* to remove cell debris and insoluble aggregates. Isolated nuclei were then washed in hypotonic buffer, lysed in solution B (3 mM EDTA, 0.2 mM EGTA, 1 mM DTT and protease and phosphatase inhibitors), and incubated on ice for 10 min. The soluble nuclear fraction and chromatin pellet were collected by centrifugation at 1,700 *g* for 4 min. The chromatin pellet was then washed in hypotonic buffer, spun down at 10,000 *g* and resuspended in nuclear extraction buffer (50 mM Tris–HCl pH 7.5, 420 mM NaCl, 10% glycerol, 1 mM EDTA, 1 mM DTT, 0.01% NP40, and protease and phosphatase inhibitors) and placed on a rotator at 4 °C for 45 min. 1 mM CaCl_2_ and 2 μl (0.4 U) of micrococcal nuclease (Sigma-Aldrich) were added and the mixture was incubated at 37 °C for 5 min with gentle shaking. EGTA (1 mM) was then added to inhibit the nuclease and the solubilized chromatin was collected by centrifugation at 10,000 *g* for 10 min.

### siRNA treatment

The control (5′-UAAUGUAUUGGAACGCAUA-3′) and BLM (5′-GGAGCACAUCUGUAAAUUA-3′) siRNAs^46^ were purchased from Eurofins, and the MUS81 siRNAs 1-2 (5′-CAGCCCUGGUGGAUCGAUA-3′ and 5′-CAUUAAGUGUGGGCGUCUA-3′) (ref. 20) were purchased from Dharmacon. One day before transfection, 6 × 10^5^ cells were seeded in 6-cm cell culture plates. In all experiments, cells were transfected with 50 nM of siRNA twice (24 h apart) using Lipofectamine RNAiMAX (Life Technologies). Proteins were analysed by western blotting 48 h after the second transfection.

### Clonogenic survival assay

For drug sensitivity tests, ~600 cells were seeded in six-well plates. After 2 days, cells were untreated or treated with hydroxyurea, camptothecin or cisplatin for 24 h (three wells per condition), washed with PBS and maintained in fresh media for 12–14 days to allow colony formation. For survival assays after siRNA treatment, cells were transfected with siRNA twice in 6-cm plates, and 24 h after the second transfection, the cells were collected and re-plated in 6-well plates (~600 cells per well, three wells per condition) and incubated for 12–14 days to allow colony formation. Colonies were stained for ~5 min with 40 mg ml^−1^ crystal violet solution (Sigma-Aldrich) containing 20% ethanol. The percent survival was calculated against untreated cells or the control siRNA sample.

### SCE analysis

Cells expressing GEN1 (tetracycline induction, 48 h) were treated with or without cisplatin (2 μM) for 1 h. The culture media was then changed and BrdU (100 μM) was added. Cells were grown for further 48 h and treated with Colcemid (0.2 μg ml^−1^) for 1 h before being collected. Metaphase chromosomes were prepared and assayed for SCEs as previously described^20^.

### Analysis of chromosome aberrations

To analyse chromosome aberrations, GEN1 was expressed from a tetracycline-inducible promoter, and the indicated proteins were knocked down by siRNA treatment (2 × , 24 h apart). At a time (8 h) after the second siRNA transfection, cells were treated with cisplatin (2 μM) for 1 h. The culture media were changed and cells were grown for further 48 h, before being treated with Colcemid (0.2 μg ml^−1^) for 1 h and then collected. Metaphase chromosomes were prepared as above.

## Additional information

**How to cite this article**: Chan, Y. W. and West, S. C. Spatial control of the GEN1 Holliday junction resolvase ensures genome stability. *Nat. Commun*. 5:4844 doi: 10.1038/ncomms5844 (2014).

## Supplementary Material

Supplementary Information Supplementary Figures 1-5
