# Supplementary material for: Spatial control of the GEN1 Holliday junction resolvase ensures genome stability
Source: Nat Commun. 2014 Sep 11;5:4844. doi: 10.1038/ncomms5844 (PMC4172962; doi:10.1038/ncomms5844)
Supplement: Supplementary Information — Supplementary Figures 1-5 [file ncomms5844-s1.pdf]

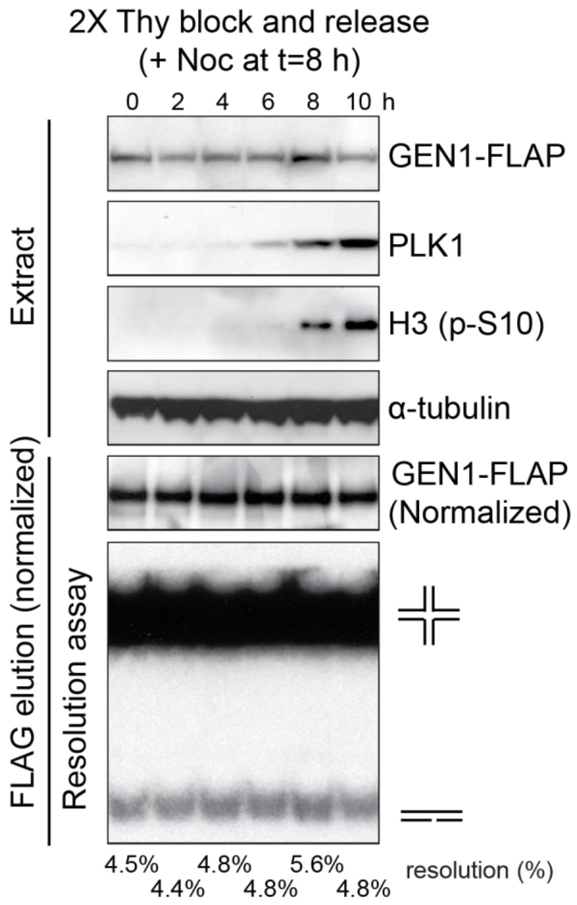

Supplementary Figure 1: Analysis of GEN1 activity throughout the cell cycle. Extracts were prepared from HeLa cells expressing GEN1-FLAP after synchronization with a double thymidine block (16 h each, 8 h in between) and release. 8 h after the 2nd thymidine release, nocodazole was added to block the cells in mitosis, as indicated. Cell extracts were analyzed by western blotting for the indicated proteins. GEN1-FLAP was affinity purified from each sample using anti-FLAG M2 agarose beads. GEN1 was then eluted by 0.5 mg/mL 3xFLAG peptides in lysis buffer. The concentrations of eluted GEN1 were normalized such that roughly the same amount of GEN1 from each sample was assayed for HJ resolution activity.

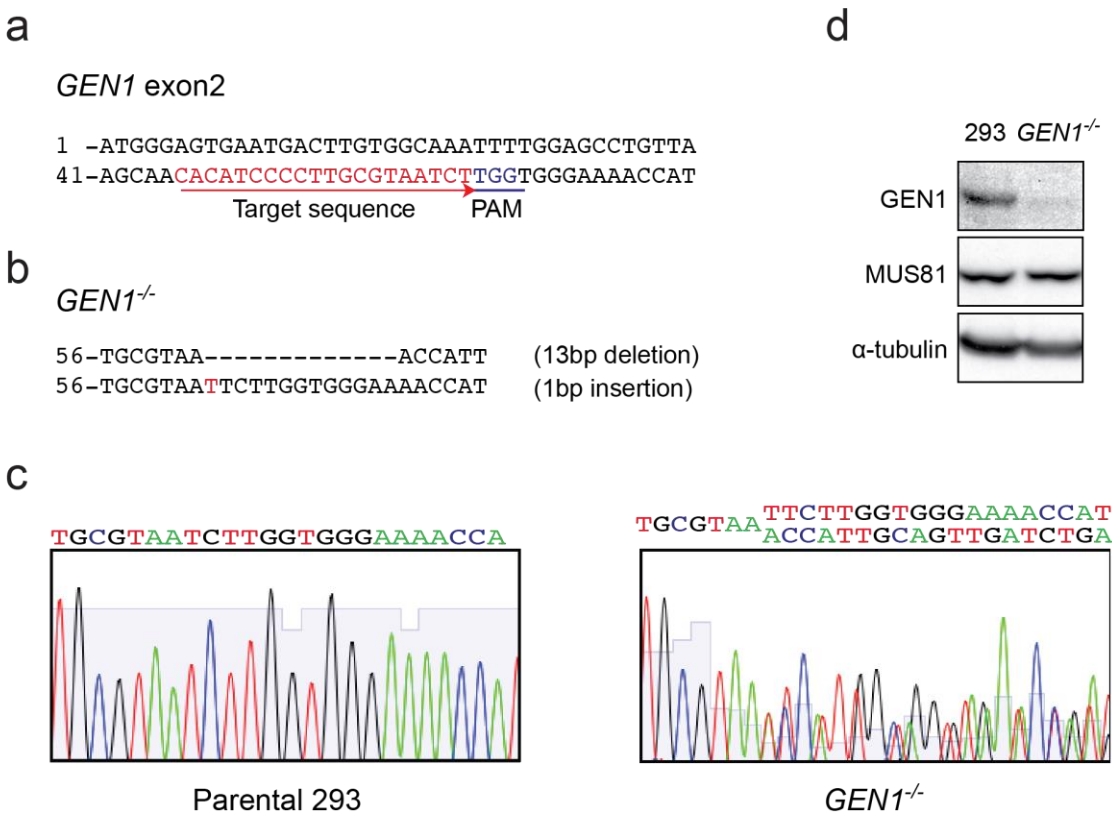

Supplementary Figure 2: Generation of GEN1 knock out cell line. (**a**) Sequence of the first 80-bp of *GEN1* exon 2 is shown. The 20-nt target sequence of the sgRNA for the plus strand of *GEN1* locus is labeled in red. The protospacer-adjacent motif (PAM) is labeled in blue. (**b**) Sequences (plus strand of each allele) of the *GEN1* locus of the *GEN1*$^{-/-}$ cells generated from Flp-In T-Rex 293 cell are shown. The dash indicates base deletion and red indicates base insertion. (**c**) Sequence chromatograms of the PCR products of the parental Flp-In T-Rex 293 cells (left panel) and the *GEN1*$^{-/-}$ cells (right panel), respectively. (**d**) Western blotting analysis of the parental Flp-In T-Rex 293 cells and the *GEN1*$^{-/-}$ cells.

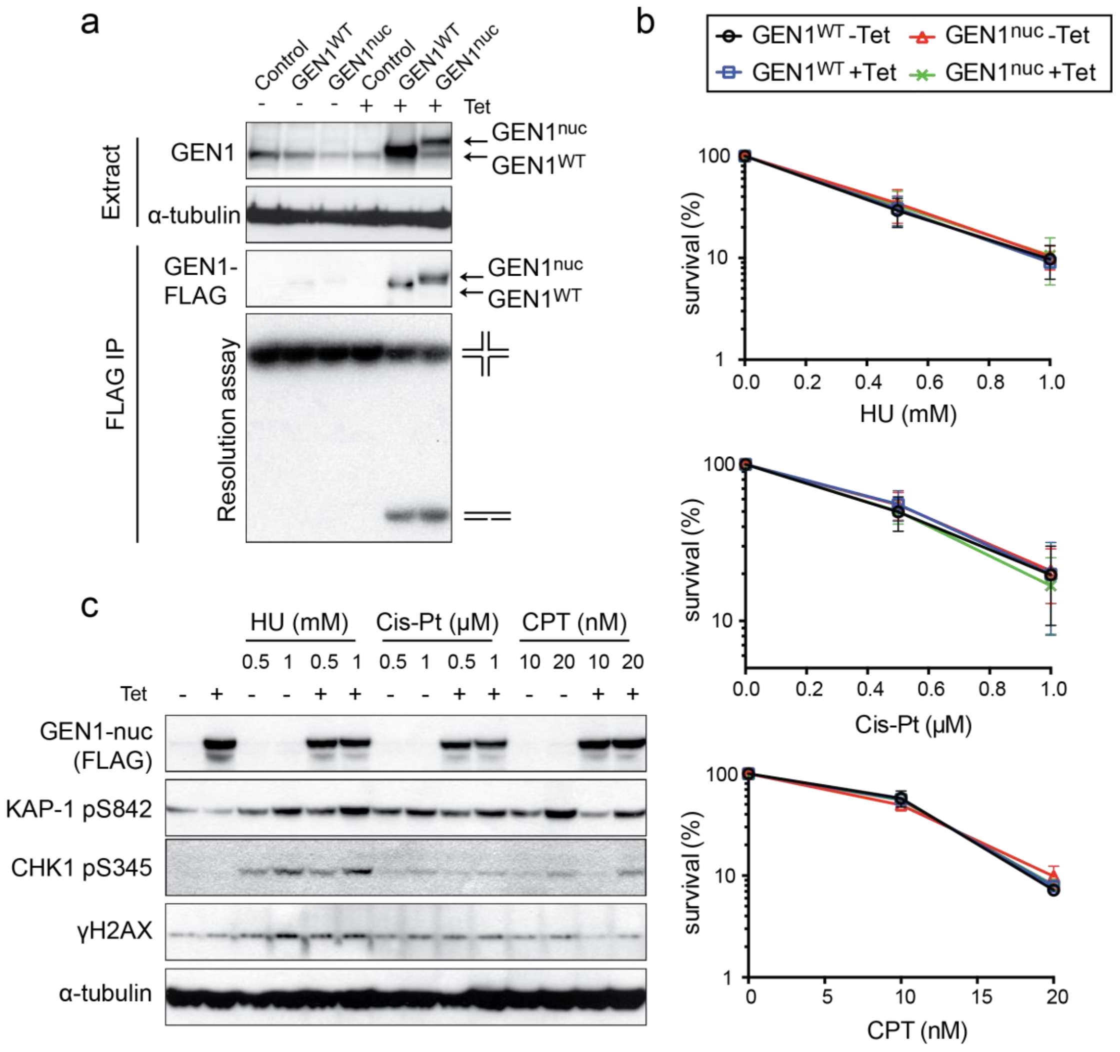

Supplementary Figure 3: Cellular expression of GEN1^{nuc} does not confer DNA damage sensitivity. (**a**) Flp-In T-REx 293 cells, and those expressing GEN1^{WT} or GEN1^{nuc}, with or without tetracyclin (Tet) induction (48 h), were grown and extracts prepared for analysis by western blotting. GEN1-FLAG was affinity-purified from each sample and assayed for HJ resolution activity. (**b**) Clonogenic cell survival assays with Flp-In T-REx 293 cells following expression of GEN1^{WT} or GEN1^{nuc} (±Tet) and treatment with the indicated concentrations of hydroxyurea (HU), cisplatin (Cis-Pt) or camptothecin (CPT). The data represent the mean ± s.d. of at least three independent experiments. (**c**) Cells expressing GEN1^{nuc} were incubated with or without Tet for 48 h, prior to treatment with the indicated concentrations of HU, Cis-Pt or CPT for 24 h. Extracts were prepared and analyzed by western blotting for the indicated proteins.

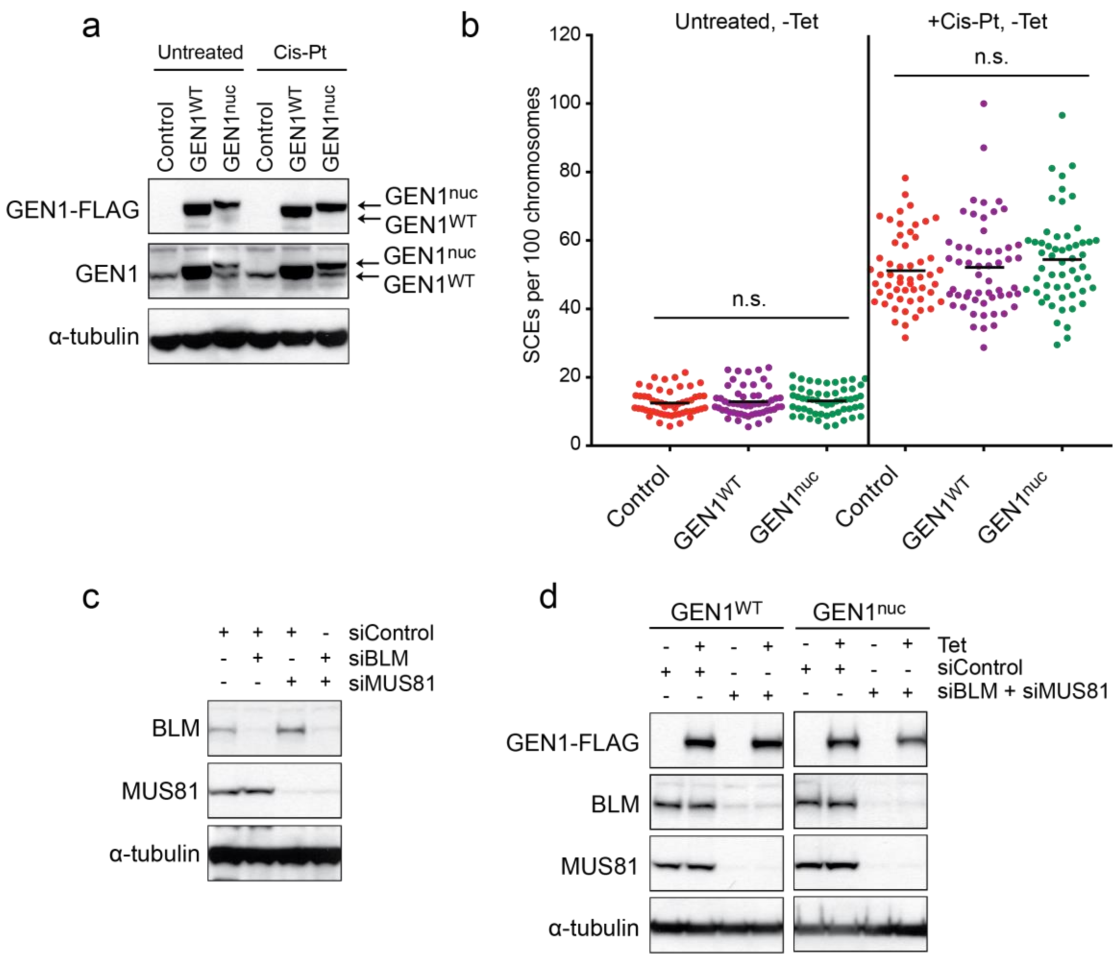

Supplementary Figure 4: Western blot and SCE analysis of cells with or without expression of GEN1 proteins. (**a**) Flp-In T-REx 293 cells, and those expressing GEN1^{WT} or GEN1^{nuc}, were treated with or without Cis-Pt (2 μM) for 1 h. The cells were then released into fresh medium for 48 h. Extracts were then prepared and analyzed by western blotting for the indicated proteins. (**b**) Flp-In T-REx 293 cells (-Tet) were treated with or without Cis-Pt (2 μM) for 1 h. SCE levels were quantified. Each data point represents a single metaphase. 55 metaphase cells (>2500 chromosomes) were counted per condition. Black bars represent the mean number of SCEs per 100 chromosomes per spread. *P* values were determined using a two-tailed t test. (**c**) Flp-In T-REx 293 cells were treated with the indicated siRNAs, and extracts were prepared and analyzed by western blotting for the indicated proteins. (**d**) Flp-In T-REx 293 cells expressing GEN1^{WT} or GEN1^{nuc} (±Tet) were treated with the indicated siRNAs, and extracts were prepared and analyzed by western blotting for the indicated proteins.

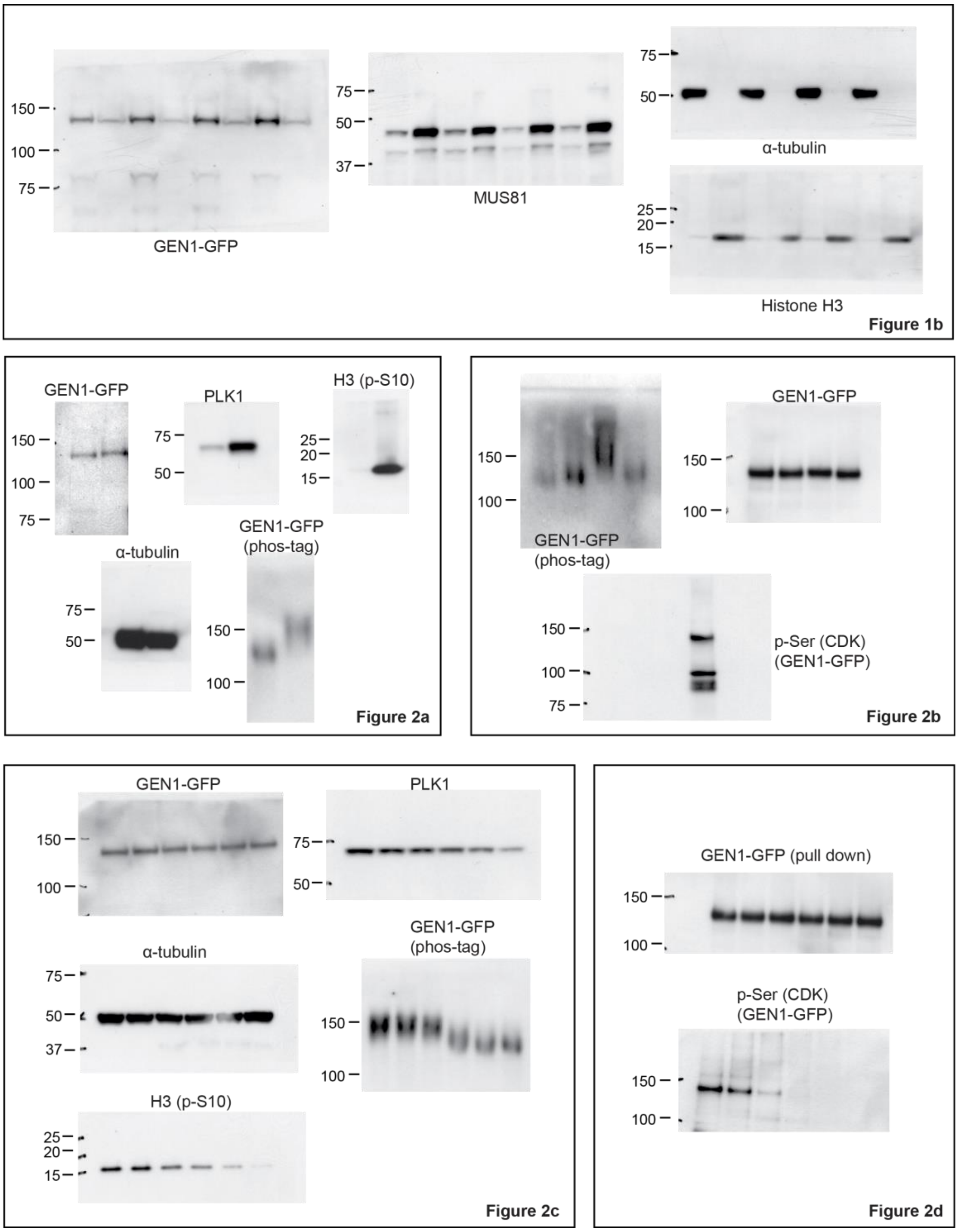

Supplementary Figure 5: Uncropped western blots of data shown in Figures 1 and 2.

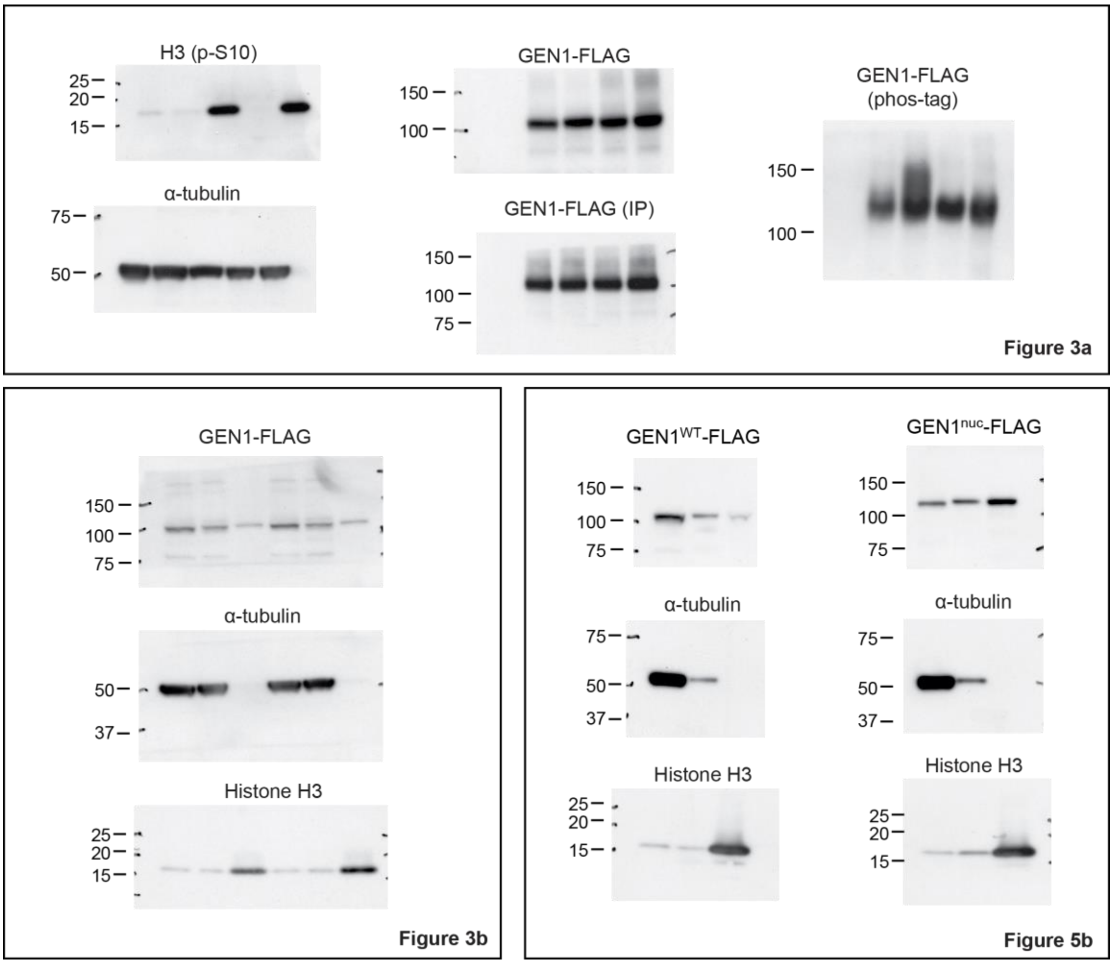

Supplementary Figure 5 (continued): Uncropped western blots of data shown in Figures 3 and 5.
